# A short - time effect of one-session application of Proprioceptive Neuromuscular Facilitation (PNF) bilateral leg pattern used with contract relax technique and asymmetrical breathing in girls with Adolescent Idiopathic Scoliosis (AIS)

**DOI:** 10.1186/1748-7161-9-S1-O63

**Published:** 2014-12-04

**Authors:** Stępień Agnieszka, Graff Krzysztof, Podgurniak Małgorzata

**Affiliations:** 1 Józef Piłsudski University of Physical Education, Warsaw, Poland; 2 Warsaw University of Life Science, Warsaw, Poland

## Background

Physiotherapy is a part of conservative scoliosis treatment [1]. Different theories, methods and exercises was proposed and used to date, but only a few of them was proved and recognized as effective. PNF is one of methods in physiotherapy used in orthopedic and neurological diseases [2]. PNF philosophy, three- dimensional patterns, techniques, breathing and movement stimulation can be useful in AIS therapy. Recent author's studies established limited range of trunk and pelvis rotation (TPR) in AIS girls with double curve scoliosis [3].

## Aim

The aim of the study was to estimate a short time effect of one- session application of PNF bilateral leg patterns used with Contract - Relax technique and asymmetrical breathing on the angle of trunk rotation (ATR) and TPR in AIS girls.

## Design

Case series.

## Methods

25 girls (12,2) with double curve scoliosis participated in the study. The right thoracic curve (28,1) and the left lumbar curve (24,6) at the spine radiography were including criteria. The clinical assessment, performed before and after therapy, comprised the Angle of Trunk Rotation (ATR) and an original test - Trunk/Pelvis/Hip Angle test (TPHA) created to evaluate TPR. PNF bilateral leg pattern, applied in the supine position with the thorax stabilization, combined with Contract Relax technique and asymmetrical breathing, was used to improve range of rotation into the direction of limitation. To determine statistical differences paired t-test/sign test/signed range test and two samples t-test/Mann-Whitney (Wilcoxon) were used.

## Results

Significant difference between right and left TPR was observed before treatment in AIS girls (p<0,001). There was no significant difference TPR after therapy. ATR values were significant lower after therapy - ATR Th (p<0,001), ATR L (p<0,01).

## Conclusion

PNF bilateral leg pattern used with Contract Relax technique and asymmetrical breathing influence ATR and TPR in AIS girls. There is necessary to continue study in numerous AIS group with different types of spine deformation.
